# ECG signal feature extraction trends in methods and applications

**DOI:** 10.1186/s12938-023-01075-1

**Published:** 2023-03-08

**Authors:** Anupreet Kaur Singh, Sridhar Krishnan

**Affiliations:** Department of Electrical, Computer and Biomedical Engineering, Toronto Metropolitan University, Toronto, ON Canada

**Keywords:** ECG, Feature extraction, Digital health, Telehealth, Signal analysis, Artificial intelligence

## Abstract

Signal analysis is a domain which is an amalgamation of different processes coming together to form robust pipelines for the automation of data analysis. When applied to the medical world, physiological signals are used. It is becoming increasingly common in today’s day and age to be working with very large datasets, on the scale of having thousands of features. This is largely due to the fact that the acquisition of biomedical signals can be taken over multi-hour timeframes, which is another challenge to solve in and of itself. This paper will focus on the electrocardiogram (ECG) signal specifically, and common feature extraction techniques used for digital health and artificial intelligence (AI) applications. Feature extraction is a vital step of biomedical signal analysis. The basic goal of feature extraction is for signal dimensionality reduction and data compaction. In simple terms, this would allow one to represent data with a smaller subset of features; these features could then later be leveraged to be used more efficiently for machine learning and deep learning models for applications, such as classification, detection, and automated applications. In addition, the redundant data in the overall dataset is filtered out as the data is reduced during feature extraction. In this review, we cover ECG signal processing and feature extraction in the time domain, frequency domain, time–frequency domain, decomposition, and sparse domain. We also provide pseudocode for the methods discussed so that they can be replicated by practitioners and researchers in their specific areas of biomedical work. Furthermore, we discuss deep features, and machine learning integration, to complete the overall pipeline design for signal analysis. Finally, we discuss future work that can be innovated upon in the feature extraction domain for ECG signal analysis.

## Background

Signal analysis is a domain which is an amalgamation of different processes coming together to form robust pipelines for the automation of data analysis. The processes for the signal analysis pipeline would be as follows:

- Data acquisition
- Data pre-processing
- Feature extraction
- Feature selection
- Model training and classification
- Performance evaluation

When applied to the medical world, physiological signals are used. This paper will focus on the electrocardiogram (ECG) signal specifically and a review of common feature extraction techniques used in the industry.

The ECG was discovered by Willem Einthoven in 1902. The ECG signal measures the electrical activity of the heart, essentially performing an electrical tracing of the heart [1]. The heart has two atria (right and left) which perform blood collection, and two ventricles (right and left) which pump the oxygenated blood to the rest of the body. The heart contracts due to electrical activity, which manifests in the ECG signal that we analyze. The ECG is the most commonly used signal in the healthcare domain for analyzing heart and overall patient health.

Acquisition of the ECG is fairly straightforward and non-invasive; surface electrodes are used on the limbs and/or the chest. Traditionally, a 12-lead ECG is taken (split into limb leads and precordial leads), but for more modern applications, single-lead ECGs are becoming more desirable and commonplace due to the reduction of complexity and data. This can be applied to the Internet-of-things (IoT) and connected healthcare domain, where telehealth is of popular concern [1]. Multi-lead ECGs are used more in clinical settings because it is the gold standard; single-lead/reduced-lead ECG signals are not typically accepted in the primary healthcare and clinical workspaces. However, single-lead/reduced-lead ECG analysis is accepted in the wellness space and ancillary healthcare systems by patients that want to track fitness and wellness to transform their lifestyles; this further facilitates the movement toward patient-centered healthcare.

**Fig. 1:**
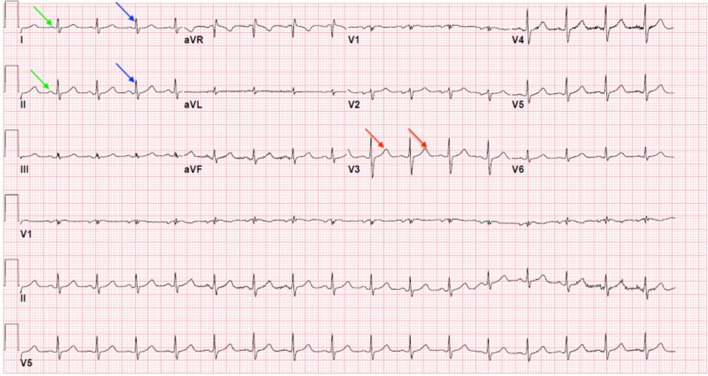
Typical ECG displaying the PQRST components. Green arrows indicate the P waves, blue arrows represent the QRS complexes, and red arrows represent the T waves [1]

Typically, the peak of the ECG ranges around 1 mV. It follows a characteristic PQRST wave pattern, as shown in Fig. 1. This is a periodic pattern repeated throughout characteristic ECG waves. Due to the commonality of the wave shape, physicians can oftentimes extract information visually from the ECG due to the morphological shape they may observe. Hence, if the wave-shape does not follow the healthy, expected morphology, it can be deduced that a cardiovascular disease is causing the anomaly (classification of the signal as either normal or pathological). Common uses of the ECG range from diagnosis of chest pain, tachycardia, bradycardia, hypertension, hypotension, myocardial injury, rheumatic heart disease, and more.

There are many features and attributes of the ECG signal that can be measured without the use of overly complex feature extraction algorithms, such as the heart rate and rhythm, PR interval, the ST segment, the QT segment, and the U, J, P, R, S, T waves just to name a few (Refer to Fig. 1 displaying a typical ECG and the PQRST components). This paper will delve into some of the more complex techniques as well.

### Evolution of feature extraction methods

Feature extraction is a vital step of biomedical signal analysis. It is becoming increasingly common in today’s day and age to be working with very large datasets, on the scale of having thousands of features. This is largely due to the fact that acquisition of biomedical signals can be taken over multi-hour timeframes, which is another challenge to solve in and of itself [2].

There are some basics to understand about physiological signal properties [3]. Signals are:

- Non-stationary
- Non-linear
- Non-Gaussian
- Non-short form

This complicates the overall feature extraction and signal analysis process even further [2, 3].

The basic goal of feature extraction is for dimensionality reduction and data compaction; in layperson’s terms, this would allow one to represent their data with a smaller subset of features; these features could then later be leveraged to be used more efficiently for ML and AI models for applications, such as classification and diagnosis. In addition, the redundant data in the overall dataset is filtered out as the information of interest is only extracted during feature extraction [2].

Useful features that are extracted from the signal should be able to represent the signal accurately, in terms of either specific patterns or behaviors observed in the signal itself. Note that before feature extraction can begin, the original signals must be made to be discrete from continuous analog signals to discrete digital signals using an analog-to-digital converter (ADC). This allows for the identification of patterns over discrete time intervals [4].

After feature extraction, typically feature selection is performed. The features selected for training the ML models can greatly affect the performance of the model, either negatively or positively. For example, if inappropriate/inefficient features are chosen to train the model, which overall does not represent the underlying signals very well, the performance of the model would degrade. A good rule of thumb is to choose application-dependent features to represent your signal versus generic features; this would ensure that the features would capture the patterns and behaviors of interest [2, 4].

Overall, feature extraction and feature selection saves on hardware and software resources, computational time, and reduces complexity, all of which can be used to apply to the world of ML and AI-based connected healthcare and telehealth [3].

**Fig. 2:**
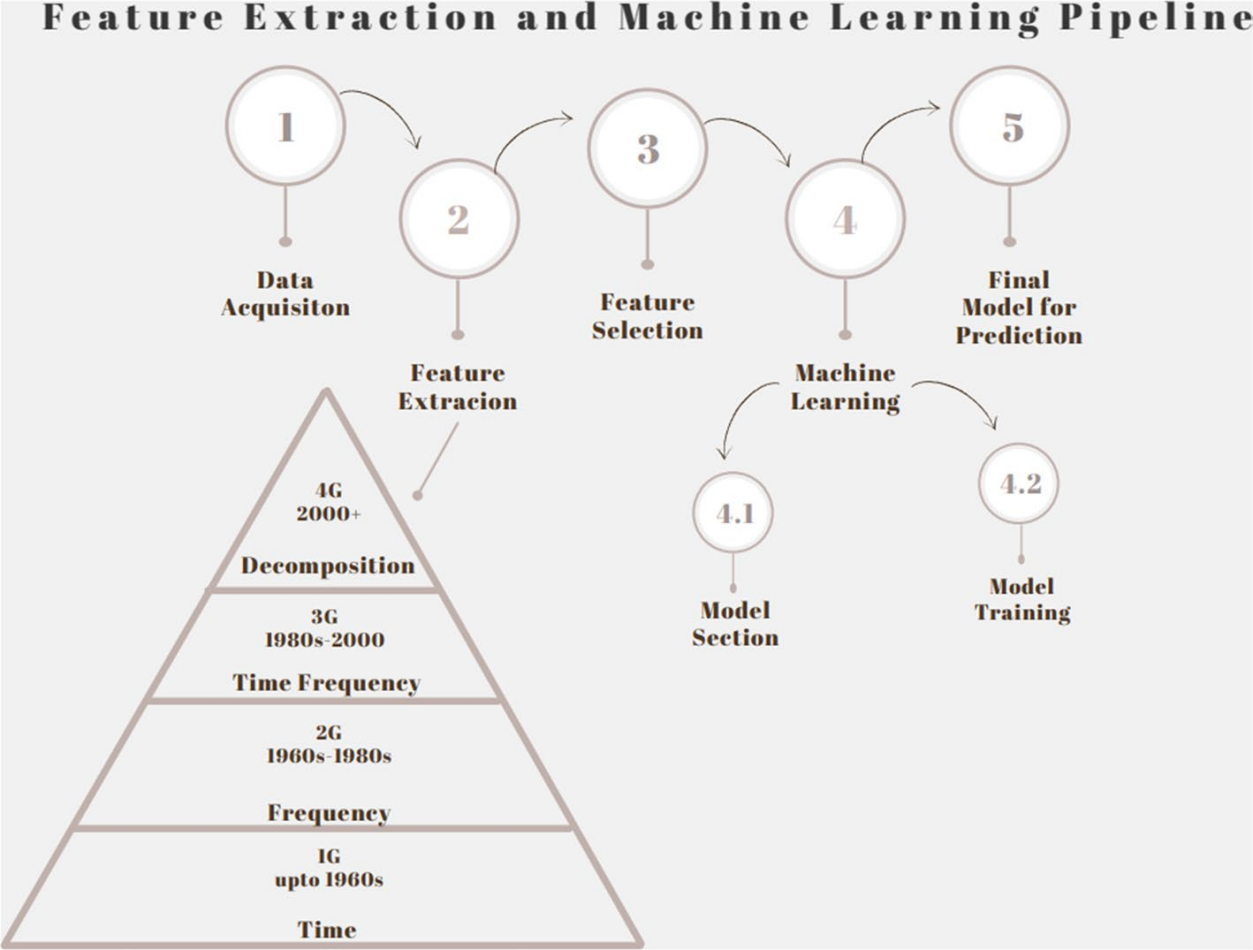
Basic feature extraction and machine learning pipeline showing the evolution of biomedical signal feature extraction techniques over the decades [3]

In this paper, we will review common feature extraction methodologies that have been applied to ECG signals over the years (refer to Fig. 2), everywhere from single-lead ECGs to multi-lead ECGs (note that the number of leads used affects the complexity of the techniques discussed, and thus result in very different feature extraction approaches). This will be organized by generation of the signal processing and feature extraction techniques. At a high level, we will go through the following (refer to Fig. 2):

- Time domain
- Frequency/Spectral domain
- Time–Frequency domain
- Decomposition domain
- Deep features

Please note that the methods discussed in this paper are by no means an exhaustive list; it is simply meant to provide a starting ground for analysis of ECG signals, and popular analysis techniques adopted in the biomedical engineering domain. This study will also go into the overall design of an ML model for biomedical signal analysis. The review work has been organized as follows: In “Significance of Features for Machine Learning” Section, the design of ML models for classification applications following successful feature extraction will be discussed. “Time-domain Feature Extraction, Frequency-domain Feature Extraction, Joint Time-Frequency Domain Feature Extraction, Decomposition Domain Feature Extraction and Deep Learning” Sections will discuss common feature extraction techniques and their advantages/disadvantages, as well as potential applications in the realm of ECG analysis. Finally, in “Discussions, Conclusions, and Future Works” Section, we will conclude the review with critical discussions, as well as potential guides toward future work.

### Search strategy for review

The publications chosen for this review were chosen based on their pedagogical relevancy for biomedical engineering students, pertaining to biomedical signal analysis. The underlying purpose of this review is for budding biomedical engineers (interested in the signal analysis domain) to have a quick reference for feature extraction algorithms that are directly correlated with biomedical applications. Hence, each review was selected based on the following eligibility criteria:

1. Relevant feature extraction technique
2. Application of algorithmic signal analysis pipeline
3. Digital/AI/Telehealth Biomedical Application
4. Any year of publication
5. Language: English

Please note that an official review protocol, as defined by the Preferred Reporting Items for Systematic Reviews and Meta-Analysis Protocols (PRISMA), does not exist. To identify potentially relevant articles, the Toronto Metropolitan University (TMU) library database was searched. The search strategy was refined through the target feature extraction method.

## Significance of features for machine learning

The natural next step after feature extraction is to apply the features to an ML model that can be used for a variety of applications, such as classifying cardiac arrhythmias. ML is a subset of the overall artificial intelligence domain. ML can help with optimizing the features used as well; the developer can identify which features have a larger/smaller positive/negative effect on the model, and use that information to optimize the overall pipeline [4].

It is important to take into account the application/problem that one wants to solve when choosing the appropriate ML algorithm to implement. Some models are more robust than others for specific applications. Some are more computationally extensive. All angles must be considered when making a decision. General criteria to follow when choosing an ML algorithm are the following: (1) Type of bio-signal, (2) Size of Feature Matrix, and (3) Availability of labeled data, just to name a few [4].

As the developer, you may also choose to evaluate more than one model for your application and select the model with the highest performance. Refer to Fig. 2 for a simple end-to-end feature extraction ML pipeline.

ML can either be supervised or unsupervised. Supervised learning refers to when the data is labeled by domain experts in the field. Most of the case studies evaluated in this paper use supervised learning as they had access to annotation files from the databases used. The labels act as ground truth for the model to learn from during the training process. Unsupervised learning is the opposite, and refers to a situation where you do not have expert labeled data. Instead, the algorithm works to find patterns in the data that are likely to distinguish between different classes. There are issues with unsupervised learning methods when working with biomedical signal data though. Since biomedical signals are better analyzed in short-duration segments, labels are applied to individual segments in supervised learning. In unsupervised learning, the ML-predicted label would be applied to the full-duration signal, which is not desirable if there are regions-of-interest that need local feature extraction applied, not global [4].

Different sets of features may be better together, so it truly is a lengthy process to find a combination that works best for the problem you are trying to solve. The reader should be aware that the number of appropriate features is also a key point of consideration; this can lead to either over-fitting or under-fitting issues.

## Time-domain feature extraction

The first generation of feature extraction was encompassed by the time domain, which is when the biomedical signals in question are analyzed with respect to time. Time-domain features allow us to quantify how the ECG signal changes over time. Typically, windowing and segmenting the signal of interest is desired for time domain analysis; this allows for the time domain features to be extracted per window. This is done because ECG signals, like other physiological signals, are non-linear and non-stationary in nature [4–6]. There are various feature extraction techniques and methods available for time domain analysis.

### Statistical features

Extracting statistical features from ECG signals is by far the least complex of the time domain feature extraction techniques. Using statistical mathematics programming languages, it becomes even simpler to implement with the use of native, built-in functions. Statistical analysis/feature extraction is not considered fiducial because knowledge of the actual ECG characteristics is not needed [7].

One popular application of statistical features can be applied for is for the use of subject recognition using ECG as a biometric trait. The feature extraction is what provides the subject-unique biomarkers that can be used to differentiate between the subjects and their ECG signals [7].

A few popular statistical features that can be extracted from the ECG are as follows:

- Mean
- Standard deviation
- Median
- Maximum value
- Minimum value
- Range
- Interquartile range
- Interquartile first quarter (Q1)
- Interquartile third quarter (Q3)
- Kurtosis
- Skewness of ECG signal

The mean and the median features can be used to measure the central tendency of the ECG signal. The statistical dispersion of the ECG is captured by the standard deviation, range, and interquartile range features. The kurtosis and skewness features are typically used to measure the asymmetry and the sharpness of the peak of the ECG signal distribution [7].

**Fig. 3:**
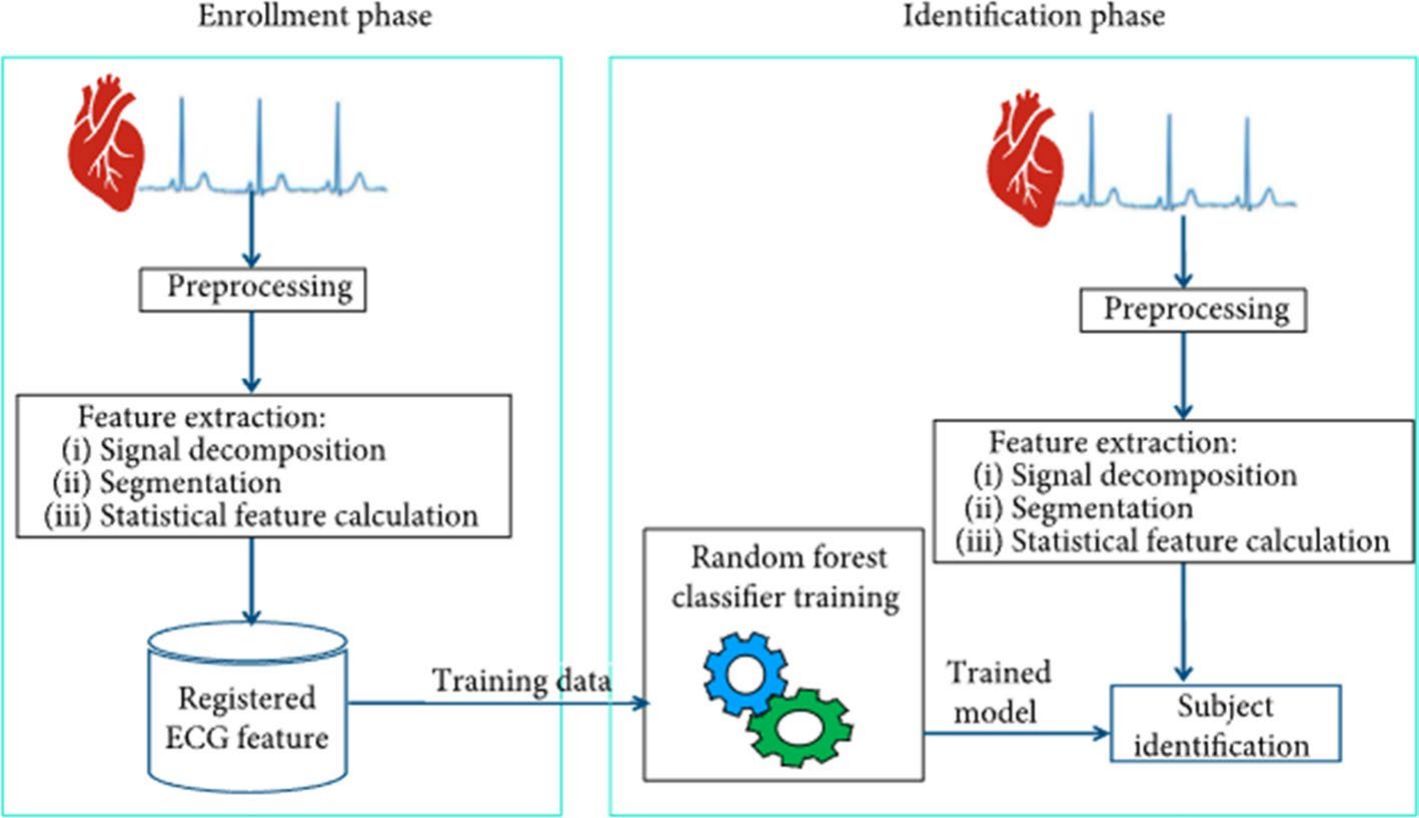
Proposed subject identification system using the statistical features from the ECG [7]

A non-linear dimensionality reduction technique, like the t-distribution stochastic neighbor embedding (t-SNE) algorithm, can be used to show that these statistical features are in fact separable, which allows for accurate and precise subject identification. Refer to Fig. 3 for the statistical feature extraction pipeline [7].

**Table:** 

**Algorithm 1:** Statistical feature extraction	
1. **Result**: Feature table with extracted features from ECG signal	
2. Import collected ECG signal	
3. Preprocess ECG signal	
a. Filter	
b. Segment into window size of choice	
4. Extract the statistical features per window	
a. Example with mean	
i. For *i* = 1:number of windows	
1. mean_*i* = mean(window_i)	
5. Assemble the feature table to be used further in machine learning algorithms for classification	

Another statistical technique that can be employed for the feature extraction from ECG signals is the principal component analysis (PCA) technique. The PCA technique is also known as the discrete Karhunen–Loève transform and the Hotelling transform. The goal of this technique is to extract the “principal components” of the signal, which are derived as a linear combination of the variables of the data (in this case, time samples of the ECG), with weights to ensure the components are mutually uncorrelated. This can be used to track temporal changes due to myocardial ischemia or signal separation during atrial fibrillation, just to name a few applications [8].

**Table:** 

**Algorithm 2:** PCA [8]	
1. **Result**: Principal components of the ECG signal	
2. Import collected ECG signal	
3. Preprocess ECG signal	
a. Filter	
b. Segment into window size of choice	
4. Principal Component Calculation:	
a. For *i* = 1:number of windows	
i. Signal-mean(window_i)	

### Multivariate autoregressive (MAR) and scalar autoregressive (SAR) modeling

Autoregressive models use the current and past values of a discretized signal to calculate the future values. This technique is conducive for data compression/compaction and reducing signal noise. This method has been applied to two-lead ECG signals, as it has been shown that the two-lead signals optimize the classification results versus one-lead ECG signals [9]. MAR models and SAR models can be used, each having its own benefits and appropriate applications. MAR has been popularly used to model heart rate and blood pressure, but not for the application of classifying cardiac arrhythmias. SAR has been used for modeling bio-signals for analysis, and for modeling heart rate variability (HRV), and for power spectrum estimation (PSD) of ECG signals [9].

The dataset used in the case study analyzed was obtained from the MIT/BIH database, which included normal sinus rhythm (NSR), atria premature contraction (APC), premature ventricular contraction (PVC), ventricular tachycardia (VT), ventricular fibrillation (VF) and supraventricular tachycardia (SVT). The NSR, PVC and APC were sampled at a frequency of 360 Hz. The VT/’VF signals were sampled at a frequency of 250 Hz. The SVT signals were sampled at a frequency of 128 Hz. The data was sampled such that all the two-lead ECG signals in the analysis had a frequency of 250 Hz [9].

In the case study, for the purposes of classifying cardiac arrhythmias, the MAR model of feature extraction was found to be superior.

**Table:** 

**Algorithm 3:** AR Model [9]	
1. **Result**: AR model coefficients	
2. Import ECG signal	
3. Preprocess	
a. Remove the noise (respiration, wandering baseline, etc.)	
b. High-pass filter with f_c_ = 2 Hz	
4. For either SAR or MAR model, chose model order 4	
5 Model coefficients are the features	

### Pan–Tompkins algorithm

The Pan–Tompkins algorithm is a popular algorithm used for the real-time detection of ECG signal QRS complexes, and analyses slope, amplitude and width. This algorithm can be used for the detection of cardiac diseases. This is a highly reliable and accurate algorithm that is able to recognize QRS complexes [11, 12].

The dataset used in this study was from the MIT/BIH and AHA database, and consisted of 48 half-hour recordings. This came together to form 24 h of ECG 2-channel data, including the annotation channel and binary-recorded timing track channel [11].

**Table:** 

**Algorithm 4:** Pan–Tompkins Algorithm [11]	
1. **Result**: QRS Complex Detection	
2. Import the ECG signal	
3. Apply a bandpass filter to the signal to reduce noise, account for the 60 Hz and T-wave interference, and correct the baseline wander	
a. Design for a desirable 3 dB passband from about 5–12 Hz	
i. Made by cascading a low-pass filter and high-pass filter	
1. Low-pass filter	
a. F_c_ = 11 Hz	
b. Gain = 36	
2. High-pass filter	
a. F_c_ = 5 Hz	
b. Gain = 32	
c. Delay = 16 samples	
4. Apply a 5-point differentiator	
5. Apply a squaring function to each time sample of the signal	
6. Apply a moving-window integrator to the signal	

### Linear predictive coding

Linear predictive coding (LPC) is another method of time domain ECG feature extraction. It has been widely used to analyze other physiological signals, like speech signals and for the spectral analysis of heart sounds, but has also been explored for the analysis of ECG signals. Specifically, with the use of Levinson–Durbin’s linear prediction model, a residual error signal feature can be obtained. It has been found that there are a variety of fairly significant properties that show that this is an important ECG feature. The case study analyzed for this method delves deeper into the use of the residual error signal feature for arrhythmia detection, namely premature ventricular contraction (PVC) detection [13].

The dataset used for this study was taken from the MIT/BIH arrhythmia database. The sampling rate was 360 Hz. There were annotation files available for comparison to the algorithm-detected PVCs [13].

**Fig. 4:**
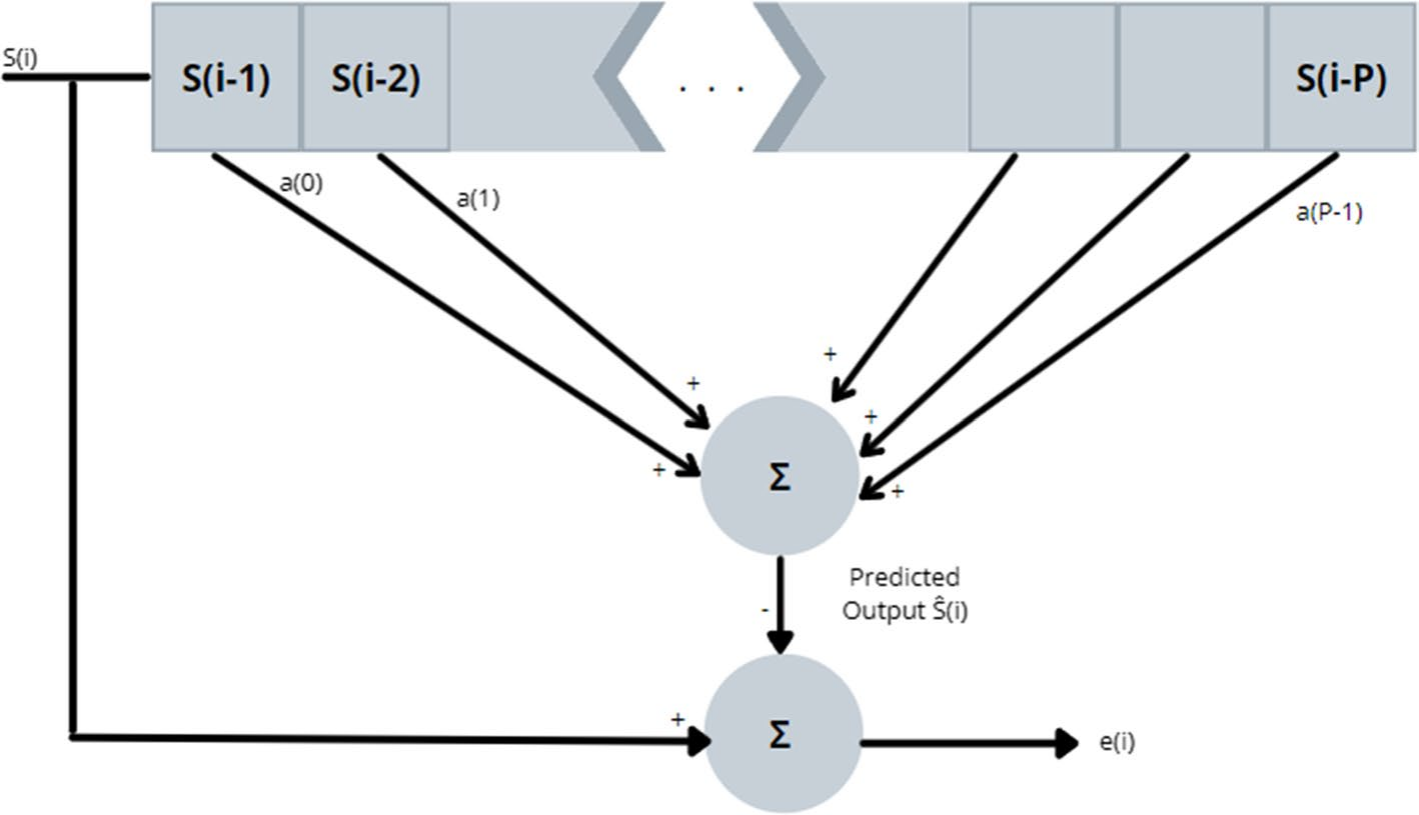
Linear Predictive Model Visualization [13]

This method is desirable as it provides accurate signal parameter estimates, and it is computationally fast. The basic premise behind LPC for ECG analysis is this: the sampled ECG signal is approximated as a linear combination of the past ECG time samples in the following way [13]:

1 $$\widehat{S}(i)={\sum }_{k=1}^{P}a\left(k\right)*S\left(i-k\right)$$

where $\widehat{S}$ is the approximation of the ECG signal, *a(k)* are the *kth* linear predictive coefficients (used as weighting factors) and *S(i-k)* are the past time sample values of the ECG signal. Refer to Fig. 4 for a visual representation of LPC [13].

**Table:** 

**Algorithm 5:** LPC Algorithm [13]	
1. **Result**: Linear predictive coefficients and the residual error signal	
2. Import signals	
3. Preprocess the ECG	
a. Noise filtering	
b. QRS detection	
i. Can use algorithm of choice, i.e., Pan-Tompkins from Sect. “Pan–Tompkins algorithm”	
4. Apply Levinson–Durbin’s Algorithm from [14]	
a. Use prediction order of P	

### Hidden Markov models

**Fig. 5:**
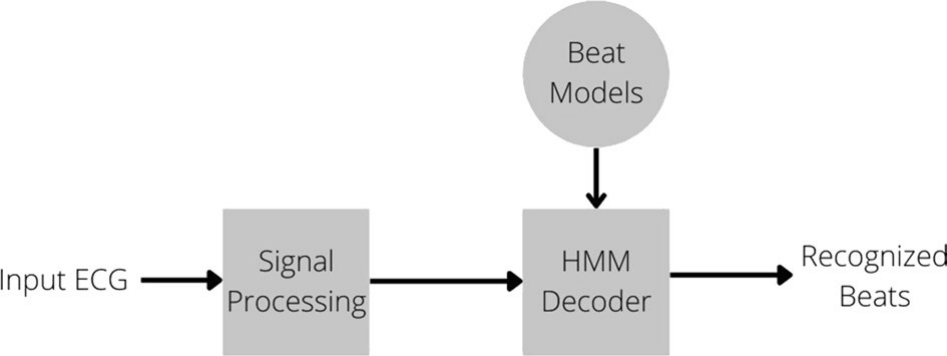
Basic process for HMM applied to ECG analysis [15]

The hidden Markov model (HMM) was first applied to ECG signals in the 1990s. Prior to this, it was mainly used for speech signals. This approach combines both statistical and structural knowledge of the ECG into a signal model. The model parameters are obtained from a maximum likelihood re-estimation algorithm. The application this case study focused on was for improved supraventricular arrhythmia analysis. The challenges posed with arrhythmia detection and classification are due to the interference from artifacts from sources, such as skeletal muscles, electrodes movement, and power-line interference. Refer to Fig. 5 for the underlying HMM process applied to ECG analysis [15].

HMM works to characterize the observed data, in this case, the ECG signal, with a probability density function (PDF). There is an underlying Markov chain that varies the PDF. The advantage with HMM is that the structural integrity is preserved for the characteristics. The goal is for the beats to be accurately identified by their wavefront components; this would allow for complete arrhythmia analysis; each waveform is assumed to correspond with the Markov process [15].

This case study proposed a “patient-dependent” arrhythmia detection technique. “Patient-dependent” simply refers to the fact that supervised training is required to analyze ECG recordings from each patient, whereas a “patient-independent” system would be able to automatically analyze any new patient ECG without supervision. Although the “patient-independent” system would’ve been more ideal, at the time the paper was written, further research was still needed in this domain [15].

The database used was from the American Heart Association (AHA) ventricular arrhythmia database. It consists of 80 1/2-h 2-channel ECG recordings which have been sampled at a frequency of 250 Hz. There was also an annotation file provided in the database [15]. Please refer to Table 1 for the summary of the time domain feature extraction methods discussed.

**Table 1:** Summary of time domain feature extraction methods for ECG

Method	Advantages	Disadvantages	Sample applications
Statistical features	Simple implementation Computationally inexpensive	Not application-specific; statistical features can be extracted for many types of data, and may not always be the best choice for physiological signals like the ECG	Biometric identification system with the use of ECG signals [7]
Multivariate AR (MAR) and scalar AR (SAR) modeling	Signal compression Improve resolution Model spectral peaks Reduce noise	SSE decreased with model order < P, but remained constant for model order ≥ 3. MAR model order 4 was chosen to extract the features More details can be incorporated into the model order, but the number of MAR coefficients and the computation for higher orders would increase rapidly Its linearity may not represent well the ECG nonstationary nature	Classify cardiac arrhythmias [9]
Pan-Tompkins	High detection accuracy even in the presence of noisy ECG signals Allows for real-time ECG analysis Does not require excessive computing power	The window size is determined empirically and thresholds depend on the accuracy of the heart rate determined in the previous segment—this can cause a domino effect of errors to occur	QRS detection, duration, amplitude and morphology for the diagnosis of cardiac diseases [10]
Linear Predictive Coding (LPC)	Correlator can be operated as an up-down counter High accuracy and fast processing Ideal for signal encoding	It has been shown that the actual LPC coefficients contain very little information for ECG signal analysis, but it is required to obtain the residual error signal feature	ECG classifications (arrhythmia detection, PVC detection, and rhythm analysis) [12]
Hidden Markov models	Preserves structural characteristics and integrity of the observed data High accuracy for low amplitude P wave detection in ambulatory ECG recordings	Not much better results than the commercial analysis systems available High computational complexity, and long analysis time (2.5 h for each 35-min AHA database tape)	Cardiac arrhythmia analysis [15]

**Table:** 

**Algorithm 6:** HMM Algorithm [15]	
1. **Result**: detection and classification of beat categories	
2. Import ECG signal	
3. Preprocess the signal	
a. Minimize artifact effects	
i. 2-Point central difference	
ii. Digital Low-Pass Filter	
4. Estimate the model parameter	
a. Use maximum likelihood estimation or the forward–backward algorithm	
5. Form the model	
6. Apply the model to detect and classify beat categories	

## Frequency-domain feature extraction

### Hilbert transform

The Hilbert Transform (HT) is defined by the following:

2 $$\widehat{x}\left(t\right)=H\left[x\left(t\right)\right]=\frac{1}{\Pi }{\int }_{-\infty }^{\infty }x\left(\Gamma \right)\frac{1}{t-\Gamma }d\Gamma$$

**Fig. 6:**
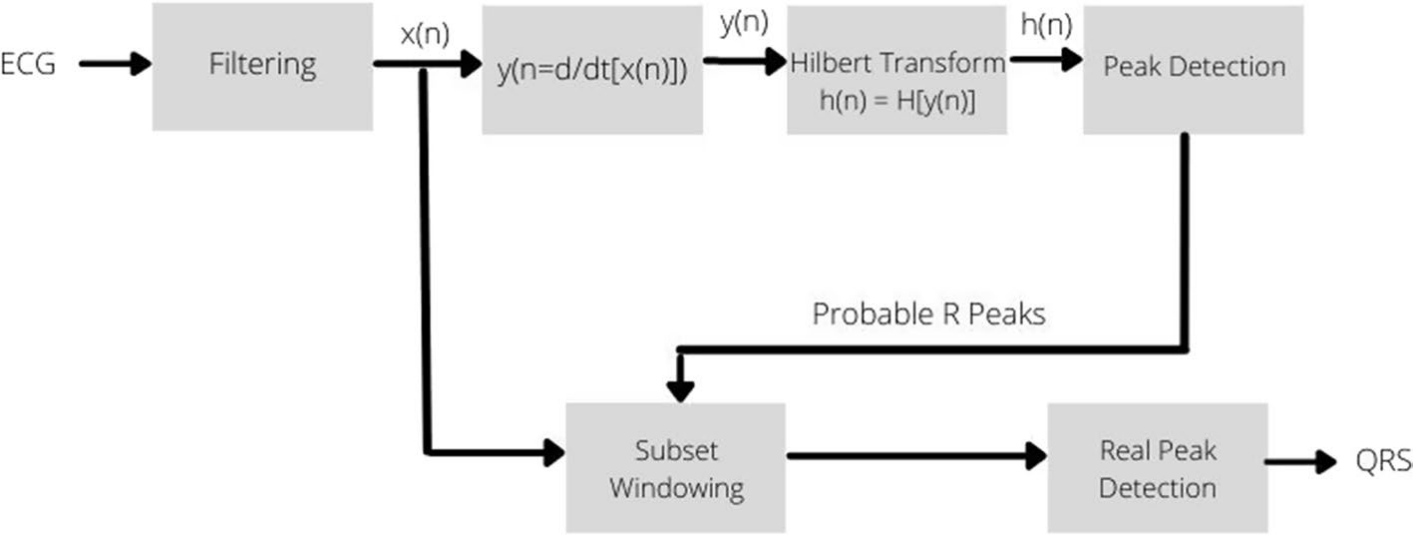
Proposed HT QRS Detection process [16]

The Fourier transform is taken of x ^(t) to move into the frequency domain. The HT is an odd function, meaning that it crosses zero whenever there is a point of inflection in the original signal. Furthermore, if a zero-cross occurs between consecutive positive and negative points of inflection in the original signal, it will present as a peak in the HT (refer to Fig. 6) [16].

These properties can be used to formulate a robust method of QRS detection from the ECG signal [16].

The dataset used in this study was from the MIT/BIH arrhythmia database. The database consisted of ECG signals recording from the modified limb lead II, as well as the modified leads V5 and V1 [16].

This method of QRS detection was very effective and accurate, in over 99% of cases, even in the presence of significant noise. However, it performed better with the modified limb lead II, versus the V5 and V1 leads. Future work is required to be able to apply this for all ECG leads/configurations [16].

**Table:** 

**Algorithm 7: Hilbert Transform Algorithm **[16]	
1. **Result**: Real-time QRS detection	
2. Import the ECG signal	
3. Preprocess the signals	
a. Bandpass FIR filter with a Kaiser–Bessel window between 8 and 20 Hz	
i. This removes the muscle noise and maximizes the QRS complexes	
b. Take the derivative of the signals	
i. This removes the base-line drift and the motion artifacts	
4. Segment the signal using 1024 points window	
5. Apply the HT	
a. Use Eq. 2	
6. Apply the Fourier Transform to the HT output	
a. Set the DC component to zero	
7. Perform peak detection using the properties of the HT	
a. Adaptive thresholding algorithm	
8. Implement a second-stage detector in parallel to confirm the peaks found by the HT algorithm	
Note: a sampling frequency of 360 Hz was used	

### Discrete Fourier Transform (DFT)

The Fourier Transform (FT) is defined by the following:

3 $$F\left( \omega \right) = \mathop \int_{ - \infty }^{\infty } f\left( t \right)e^{{\left( { - j\omega t} \right)}} dt$$

**Fig. 7:**
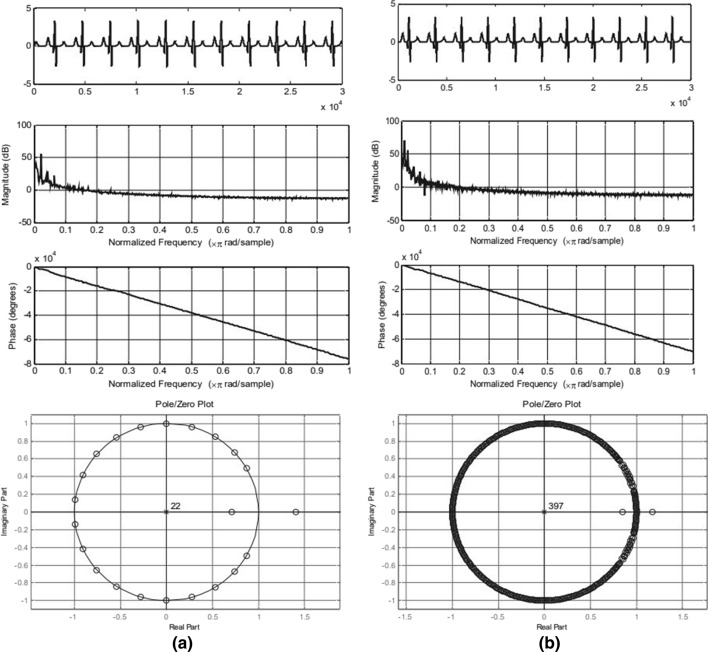
FFT and DFT analysis of a normal ECG **a** and a noisy ECG **b** [17]

The FT outputs the Fourier coefficients and it can be analyzed to better understand the underlying frequency distribution of the signal. If the signal is discrete, the DFT is used. The fast Fourier transform (FFT) is a fast and efficient implementation of the DFT. This algorithm can be used to find abnormalities in the ECG signals. Refer to Fig. 7 for the FFT and DFT analysis of a normal ECG (a) and a noisy ECG (b) [17].

**Table:** 

**Algorithm 8: FFT/DFT Algorithm **[17]	
1. **Result**: Fourier Coefficients	
2. Import the ECG	
3. Preprocess the ECG	
4. Take the FFT of the signal	

### Mel frequency cepstral coefficients (MFCC) analysis

The MFCC is a linear representation of the cosine transforms of a short duration of logarithmic power spectrum of the ECG signal. It has popularly been used for vocal analysis and recognition. A huge advantage of MFCC is that the bulb of the features of the signal is concentrated into the first few coefficients [18].

The dataset in this case study was obtained from the MIT/BIH arrhythmia database. The records are 30 min long per patient, and contain both normal and abnormal ECG signals. Ultimately this can be used to support cardiologists in the ECG classification process [18].

**Fig. 8:**
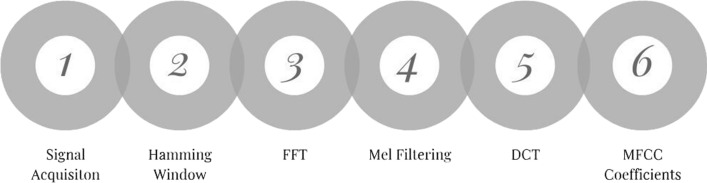
MFCC Calculation pipeline [18]

The results show that this is a very robust system and it provides quick decisions. Future work will include a deeper classification of the nature of the abnormalities detected, i.e., tachycardia, bradycardia, etc. Refer to Fig. 8 for the MFCC pipeline [18].

**Table:** 

**Algorithm 9: MFCC Algorithm **[18]	
1. **Result**: Mel Cepstrum coefficients to form a feature vector	
2. Import ECG signals	
3. Preprocess the signals	
4. Segment signal into durations on the scale of 20-30 ms	
5. Apply a Hamming window to the signal segments	
6. Apply the FFT	
a. Obtain the amplitude spectrum	
7. Filter using the “Mel Filter” which is a bank of filters pass type band triangular	
8. Apply the Discrete Cosine Transform (DCT)	

### Discrete cosine transform (DCT)

In the digital world, security is becoming a huge concern. We are also moving rapidly toward the medical-technological revolution, in which we already see everything from smartwatches to smart homes integrating seamlessly with our lives.

What if your unique ECG could be used as a biometric for user identification and authentication when walking into your home? This is quite plausible as the ECG is almost a completely unique human characteristic due to the morphology and amplitudes of cardiac complexes being controlled by individual factors. These factors range from heart size, shape and position, to the presence of possible pathologies. This is what this case study looks to solve. The biometrics in individual ECG signals are confidential, sensitive, and difficult to steal/replicate. Thus, it has great potential to be used for this purpose [19].

The algorithm proposed uses discrete cosine transform (DCT) and autocorrelation to extract features from the ECG; the effect of myocardial infarction is also taken into consideration to test if those individuals can still be recognized by the system. Essentially, the DCT coefficients would be estimated for the autocorrelated heartbeat signals [19].

The dataset used in this study was taken from the European ST-T database (healthy signals), as well as the MIT/BIH arrhythmia database (pathological signals). The signals from the European ST-T database were from 40 subjects, all healthy; each recording was taken for a duration of 1 min, and sampled at 256 Hz [19].

Overall, it was found that the biometric system proposed in this case study was able to effectively identify the subjects with a 97.5% overall identification perforation. It had a false positive rate of 0.1667, and a negative identification rate of 0.025 [19].

**Table:** 

**Algorithm 10: DCT Feature Extraction Algorithm **[19]	
1. **Result**: DCT coefficients	
2. Import the ECG Signals	
3. Preprocess the signals	
a. Butterworth band-pass filter between 1 Hz and 40 Hz	
4. Localize the highest peaks, constituted by the R peaks in the signal	
5. Perform autocorrelation to obtain 21 coefficients	
6. Perform DCT on the autocorrelation output	

### Autoregressive (AR) models

The autoregressive method of feature extraction from the frequency domain will be focused on the residual ECG (rECG). The rECG is a signal in which the ventricular components of the original ECG have been canceled out, or removed, through beat averaging techniques. It is used to extract spectral parameters from these signals to estimate the dominant atrial cycle length (DACL) obtained from patients suffering from episodes of atrial fibrillation (AF). The DACL is an important feature because it is related to atrial refractoriness, and there has been an observed increase in DACL before spontaneous termination of AF [20].

It has been found experimentally, that for patients experiencing AF, they have a main spectral component, fo, found in the range of 3–12 Hz. The DACL is the inverse of this spectral component [20].

The dataset in this study was taken from Physionet’s Spontaneous termination AF database. The rECGs were constructed for each of the recordings in the database. There is a collection of 80 two-channel ECG signals in the database, each being a recording of AF for a duration of 60 s. They have been sampled at a frequency of 128 Hz [20].

**Table:** 

**Algorithm 11: Autoregressive Algorithm (Frequency Domain) **[20]	
1. **Result**: Estimate of f_o_*/*DACL feature	
2. Import the ECG signal	
3. Preprocess the signal	
a. Construct the rECG signal through beat-to-beat subtractions of the averaged QRST complex	
4. Perform Spectral Estimation	
a. Use Welch’s periodogram to estimate the power spectral density (PSD)	
i. Use a 512-point Kaiser window with an overlap of 256 points	
5. Locate spectral peak in the range of 3–12 Hz	
a. This becomes the f_o_ estimate	
b. Use method 2 from [20]	
6. Downsample rECG series with *f*_s_ = 32 Hz	
7. Fit an AR model to each rECG series	
a. Use model order = 8	

### Eigenvector methods

The Eigenvector method is used to estimate the PSD of a noise-corrupted signal. It is based on the Eigen decomposition of the autocorrelation matrix of said noisy signals. The advantage of this method is its robustness under systems containing significant amounts of noise; even if the SNR is low, this method can produce a high-resolution spectra of the input signal, in this case ECG signals. It is best applied in the case where the ECG signals are buried in noise [8].

The main eigenvector method that will be discussed in this paper is Multiple Signal Classification (MUSIC). This method utilizes the average spectra of all the eigenvectors of the signal. These eigenvectors are related to the noise subspace. The PSD can be estimated with the following [8]:

4 $${P}_{MUSIC}\left(F\right)= \frac{1}{\frac{1}{K}({\sum }_{I=0}^{K-1}\left|{A}_{i}\left(f\right)\right|{)}^{2}}$$

where *K* is the noise subspace dimension and *A*_*i*_*(f)* is the desired polynomial. The MUSIC method has been shown to be superior for ECG analysis in the frequency domain [8]. Please refer to Table 2 for the summary of the frequency domain feature extraction methods discussed.

**Table 2:** Summary of frequency domain feature extraction methods for ECG

Method	Advantages	Disadvantages	Sample applications
Hilbert transform	Accurate Performs excellently even in the presence of significant noise in the signal	Future work required to overcome the challenges faced when using other leads with this method	QRS detection [16]
FFT and DFT	Precise detection of abnormalities in the signal Simple to implement	Challenges with capturing instantaneous frequency content Application to multi-channel signals difficult	ECG analysis and abnormality detection [17]
MFCC	High precision of calculation and decision-making High speed, can be used for real-time analysis Robust system	Highly sensitive to noise	Cardiac pathologies detection system [18]
DCT	High accuracy and simple to measure Unique to Individual Energy compression/conservation	Output is always real-valued, so quantization is needed to get integer-valued output Specify if the cosine function to be applied is odd or even	ECG analysis for user identification and authentication [19]
Autoregressive (AR)	Handles low-component, noisy signals Natural alternative to f_0_ estimate Handles case of when the intensity of the fibrillatory wave is rather small and not well concentrated in frequency	Tradeoff with the AR model order. If it is too small, the AR models extract the prevalent features of the rECG signals, among which the fibrillatory wave emerges clearly. If too big, the parametric spectrum tends to be identical to the nonparametric one, leading to overfitting results. Compromise is 7 < M < 10	Detection of atrial fibrillation from the rECG [20]
Eigenvector	Performs well even if SNR is low, therefore ideal for noisy data	MUSIC, which is identified as the superior method, causes the production of spurious zeros to occur. This can be solved by applying Minimum-Norm technique, but this increases the complexity of the algorithm	ECG analysis of signals composed of sinusoids buried in noise [8]

**Table:** 

**Algorithm 12: Eigenvector Feature Extraction Algorithm **[8]	
1. **Result**: PSD (MUSIC) to be used as a feature vector	
2. Import ECG signal	
3. Preprocess the signal	
4. Apply MUSIC formula from (4)	
5. Extract the PSD as the feature vector	

## Joint Time–Frequency domain feature extraction

Joint time–frequency analysis comes in handy when considering the non-stationarity property of ECG signals [3, 5]. Since the ECG signals are inherently non-stationary in nature, it is beneficial to represent the signals in two dimensions, with time and frequency as the coordinates [21]. In this section, we will explore various methods of time–frequency domain feature extraction of ECG signals.

Please note that the wavelet transform will be discussed in “Decomposition Domain Feature Extraction” Section of this paper, as it is seen as more of a decomposition technique. However, it does also perform time–frequency analysis (or more appropriately a joint time-scale analysis), so readers be aware of this overlap.

### Wigner–Ville distribution (WVD)

It has been argued that time–frequency analysis of ECG signals can relay and reveal more information about the signal, versus analyzing in the single domains. The WVD is one such time–frequency analysis method; it can be used for the detection of P waves in the time–frequency domain. P wave detection is important since it can directly be used for cardiac rhythm analysis [21].

The basic idea behind the WVD is this: for each time point in the ECG signal, a windowed WVD will be computed, to form a 2D “image” that is representative of the energy distribution of the underlying signal. The WVD of a signal, *s(t),* is defined as the following [21]:

5 $$W(t,f) = \int z(t+\frac{\Gamma }{2})z*(t+\frac{\Gamma }{2}){e}^{-j2\Pi f\Gamma }d\Gamma$$

where the analytic signal *z(t)* is defined as:

6 $$z\left(t\right)=s\left(t\right)+ jH\left[s\left(t\right)\right]$$

where *H[s(t)]* is the Hilbert transform of the signal.

In this particular study, the use of the cross WVD is used to cross two signals, *s*_*1*_*(t)* and *s*_*2*_*(t)* together. If *s*_*1*_*(t)* and *s*_*2*_*(t)* have similar time–frequency characteristics, the imaginary part of the cross WVD would be zero (mono-component behavior). If they have differing time–frequency characteristics, the imaginary part of the cross WVD would take non-zero values (multi-component behavior). The cross WVD is defined as [21]:

7 $$W12(t,f) = \int {z}_{1}(t+\frac{\Gamma }{2}){z}_{2}*(t+\frac{\Gamma }{2}){e}^{-j2\Pi f\Gamma }d\Gamma$$

Note that the “normal” WVD, as shown in Eq. 5, is real, while the cross WVD, shown in Eq. 7, is complex [21]. The cross-terms in the output image would be indicative of the artifacts present in the signal. Generally, the cross-terms appear during the P wave, and are represented as negative areas in the image. This can be used to detect the P waves [21].

Once the cross WVD algorithm is applied to the signal, the 2D energy distribution image is outputted; this image provides information about the modulation laws of the signal, in both the amplitude and frequency, which are important signal parameters. The dataset used in this study was taken from the MIT/BIH database [21].

**Table:** 

**Algorithm 13: Wigner–Ville Distribution Algorithm **[21]	
1. **Result**: P wave detection & WVD features	
2. Import the ECG signals	
3. Preprocess the signals	
4. Integrate the imaginary part of the cross WVD output along the frequency axis	
a. $A = Im\int {w}_{\mathrm{1,2}}(t,f)df$	
i. This can be simplified to:	
$$A={s}_{1}(t)*H[{s}_{2}(t)] - {s}_{2}(t)*H[{s}_{1}(t)]\quad \quad (8)$$	
b. This will determine the non-zero areas that detect the P waves	
5. Integrate the real part of the cross WVD along the frequency axis	
a. $E = Re\int {w}_{\mathrm{1,2}}(t,f)df$	
i. This can be simplified to:	
$$E={s}_{1}(t)*{s}_{2}(t) +H[{s}_{1}(t)]*H[{s}_{2}(t)]\quad \quad (9)$$	
6. Normalize *A* by *E* to enhance the WVD image	
Note: this algorithm avoids the computationally exhaustive WVD generation	

### Generalized tensor rank one discriminant analysis (GTR1DA)

The study that proposed the GTR1DA technique is interested in feature extraction applied to direct tensor data inputs. The ECG signals used in this study are represented by third-order tensors in the spatial time–frequency domain (12-lead ECGs converted to third-order tensors). This can help achieve greater classification accuracy than other methods [22].

The study states that there is a current issue with the methods of ECG feature extraction being explored; they mainly are applied and developed for 2-lead ECG signals, which means they cannot later be applied to 12-lead ECG signals, which are the clinical gold standard. When we use fewer leads, we are discarding much of the structural information of the ECG, and we lose spatial information as well. In theory, if all 12-leads could be considered for a feature extraction process, more robust features would be extracted, leading to a more accurate and efficient automatic analysis of the ECG signals, and the classification would be improved [22].

The tensors used in this study were constructed using the short-time Fourier transform (STFT) on the raw ECG signals. STFT is used instead of FT since it can collect temporal information about when the frequency components occur [22].

The dataset used in this study was provided by a hospital with the help of SiWei medical company and the SiWei Remote ECG diagnostic center. The entire database spans 3 years, and contains 98,287 segments (20 s each) of ECG data. The sampling rate used for this data was 500 Hz. A subset of 3000 segments was taken from this dataset to test GTR1DA, and were annotated by clinical physicians.

**Table:** 

**Algorithm 14: GTR1DA Algorithm **[22]	
1. **Result:** Class mean tensor, total mean tensor, and mean tensor of tensor pair	
2. Import the raw ECG signal	
3. Preprocess the ECG Signal	
a. Perform denoising	
b. Segment the ECG signal	
c. Perform R-peak alignment	
4. Take the short-time Fourier transform of the signal	
a. Step output: Tensor ECG data	
5. Split the Tensor ECG data into training and testing data	
6. Perform GTR1DA on the training data	
a. Calculate the class mean tensor	
b. Calculate the total mean tensor	
c. Calculate the mean tensor of tensor pair	
d. Check for convergence	
7. Form the training feature vectors	
a. Train the ML model chosen	

### Short-Time fourier transform (STFT)

The STFT can be used to compute and analyze the energy distribution of the ECG signal. It is essentially used to compute the strength of frequencies in the signal around time *t*. Features are then extracted from said energy distributions to use for classification algorithms. The STFT is defined as follows [23]:

10 $$STFT(t,f) = \int x({t}^{^{\prime}})\Upsilon*{e}^{-j2\Pi f{t}^{^{\prime}}}d{t}^{^{\prime}}$$

where *x(t)* is a finite length window, and *x(t’-t)* is the same window, but centered about time *t* [23].

The STFT has a tradeoff between time resolution and frequency resolution though, thus making the features limited by the accuracy of the frequency distribution. If the resolution in the frequency domain is increased, a longer data segment of ECG is required; however, the longer the ECG data, the higher the variation of frequency in the time domain. This means that if we want a high time resolution, we require a shorter window of ECG data [23].

The ECG dataset used in this study was taken from the Staley cardiac arrhythmia database, from which the raw data was collected by the Wisconsin-Dane County EMT-defibrillation program. It includes recordings of ventricular fibrillation, asystole, and more. The signals were acquired at a sampling rate of 100 Hz. Normal rhythms were taken from the MIT/BIH database, which are sampled at 360 Hz [23].

**Table:** 

**Algorithm 15: Short-Time Fourier Transform (STFT) **[23]	
1. **Result**: 3 features (see Step 5) for further use in classification algorithms	
2. Import the ECG Signals	
3. Preprocess the signals	
a. Bandpass filter using 2 Hz and 20 Hz as cutoff frequencies, with filter order 61 as determined using a Hamming window	
4. Perform STFT based on Eq. (10)	
5. Feature Extraction	
a. Feature 1: Frequency of maximal intensity/peak frequency—F_m_	
b. Feature 2: Normalized energy in the peak frequency band defined around F_m_	
c. Feature 3: Normalized energy in the harmonics of F_m_	

### Cone-shaped Kernel (CKD)

The CKD method was developed to reduce the cross-terms found with other time–frequency methods; it has been designed as a lateral inhibition function. This means that when the intensity computation of the signal at specified frequencies occurs, a neighborhood around that frequency will contribute positively, while frequencies outside the neighborhood contribute negatively. It also allows for an improved time–frequency resolution. The CKD is defined as the following [23, 24]:

11 $$CKD(t,f)=\int \int \varphi (t-u,\Gamma )x(u+\frac{\Gamma }{2})x*(u+\frac{\Gamma }{2}){e}^{-j2\Pi \Gamma }dud\Gamma$$

12 $$\varphi (t,\Gamma ) = g(\Gamma ), \left|\Gamma \right|\ge a\left|t\right|, 0 otherwise$$

where *x* is the original signal and *φ* is the kernel. The bounds applied to parameter *a* are the following: *2* ≥ *a* < *∞* [23].

The ECG dataset used in this study was taken from the Staley cardiac arrhythmia database, from which the raw data was collected by the Wisconsin-Dane County EMT-defibrillation program. It includes recordings of ventricular fibrillation, asystole, and more. The signals were acquired at a sampling rate of 100 Hz. Normal rhythms were taken from the MIT/BIH database, which is sampled at 360 Hz [23].

**Table:** 

**Algorithm 16: Cone-shaped Kernel (CKD) Algorithm **[23]	
1. **Result**: 3 features (see Step 5) for further use in classification algorithms	
2. Import the ECG signals	
3. Preprocess the signals	
a. Bandpass filter using 2 Hz and 20 Hz as cutoff frequencies, with filter order 61 as determined using a Hamming window	
4. Perform CKD based on Eqs. (11–12)	
5. Feature Extraction	
a. Feature 1: frequency of maximal intensity/peak frequency—*F*_m_	
b. Feature 2: normalized energy in the peak frequency band defined around *F*_m_	
c. Feature 3: normalized energy in the harmonics of F_m_	

### Choi–Williams distribution (CWD)

The CWD method was developed to reduce the cross-terms found with other time–frequency methods (namely the WVD method). It is sometimes referred to as the Reduced Interference Distribution (RID) as well. The CWD can be defined as the following [25]:

13 $${CW}_{x}\left(t,f\right)= \sqrt{\frac{2}{\pi }}{\iint }_{-\infty }^{\infty }\left(\frac{\sigma }{\left|\tau \right|}\right){e}^{\frac{2{\sigma }^{2}{\left({t}_{1}-t\right)}^{2}}{{t}^{2}}}x\left(t+\frac{\tau }{2}\right){x}^{*}\left(t-\frac{\tau }{2}\right){e}^{-j2\pi f\tau }dtd\tau$$

where the kernel function is the following:

14 $$\varphi \left(\zeta ,\tau \right)={e}^{-(\frac{{(\pi \zeta \tau )}^{2}}{{2\sigma }^{2}})}$$

$\varphi \left(\zeta ,\tau \right)$ is the parameterization function or the kernel; The kernel works as a weighting function; it attempts to keep the signal unchanged while rejecting the cross-terms [25]. If results are impacted by cross-terms, the kernel function should be leveraged to mitigate.

The dataset used in this study was taken from the MIT/BIH arrhythmia database, and it consisted of signals that were classified as the following: normal, left and right bundle branch blocks, premature ventricular contraction, paced beat, and the fusion of paces and normal beats. The method proposed achieves a classification accuracy of 99% [25]. Please refer to Table 3 for the summary of the time–frequency domain feature extraction methods discussed.

**Table 3:** Summary of time–frequency domain feature extraction methods for ECG

Method	Advantages	Disadvantages	Sample applications
Wigner-Ville distribution (WVD)	Excellent resolution for the energy distribution along both the time and frequency axes of the underlying signals WVD images show the negative areas; this provides more information about the time–frequency behavior of the ECG	WVD generation is computationally exhaustive. There are ways to combat this though as discussed in Algorithm 13 High presence of cross-terms/interference terms—it is desirable to reduce this	Detection of P waves in the time–frequency domain [21]
Tensor rank one discriminant analysis (TR1DA)	A natural way of representing ECG data, without compromising the structure information Considers the distribution characteristics of the original ECG data and of the adjacent points over the different classes GTR1DA needs less steps (about one-third of what is required with TR1DA) to convergence than the original TR1DA method	TR1DA is an extension of linear discriminant analysis (LDA), it also exhibits the same limitations. GTR1DA tries to overcome these limitations, but cannot overcome the small sample size issue, which is a result of the number of training samples being substantially smaller when compared to the larger dimensions of the feature space [22]	Auto-diagnosis of heart diseases [22]
Short-time Fourier transform (STFT)	Can detect the modal frequencies of linear time-invariant systems and their time localization very well [24] Changes in modal frequencies due to structural damage can also be detected well [24]	Trade-off between time resolution and frequency resolution, making the features limited by the accuracy of the frequency distribution. Thus, it is not possible to have both optimal time resolution and optimal frequency resolution at the same time	Arrhythmia classification [23]
Cone-shaped Kernel (CKD)	Good cross term suppression Excellent time–frequency resolution	The CKD does not satisfy the non-negative property of time–frequency distributions There is a delay in the system, which will impact the implementation of this algorithm for real-time applications	Arrhythmia classification [23]
Choi-Williams distribution (CWD)	High classification accuracy Good cross term suppression Features for each class are largely separated from the others	There is a delay in the system, which will impact the implementation of this algorithm for real-time applications	Arrhythmia detection and classification [25]

**Table:** 

**Algorithm 17: Choi-Williams Distribution Algorithm **[25]	
1. **Result**: 16 CWD features	
2. Import the ECG signals	
3. Preprocess the ECG signals	
a. Band-pass filter to remove different artifacts, such as baseline wander, muscles noise, and interference noise of 60 Hz	
4. Perform R-peak detection	
a. Segment the signal into different beats based on the R-peak detection	
b. Seven samples before the R-peak and eight samples after the R-peak (16 samples total) are time–frequency transformed using the CWD	
c. The CWD of these 16 samples become the 16 features that are extracted	

## Decomposition domain feature extraction

This section will discuss methods of decomposition for ECG signals in depth. The basic premise of all the methods discussed is as follows: decompose the ECG signal, and select the desired components, while rejecting the undesirable components. This helps with data compression as well and can be applied to the IoT and connected healthcare domain [1].

As for the sparse representation of ECG signals, it is not actually used for traditional feature extraction from ECG signals. It is more involved with the transmission and storage of the ECGs, almost as an alternative to the Nyquist theorem popularly employed in the signal analysis realm. Over the past decades, it has not picked up traction for its use as a feature extraction method, but since it is involved in transmission and storage, it can be used to augment other algorithms, especially those concerned with the IoT and connected healthcare domain [3].

### Empirical mode decomposition (EMD)

The empirical mode decomposition (EMD) method allows for the ECG signal to be split into levels of intrinsic mode functions (IMFs), correlated to the frequency distribution in the signal (from lower to higher frequencies). The IMFs are created through an interactive procedure called “sifting.” Certain requirements must be met for an IMF to truly be an IMF [26]:

1. The count of local extrema, as well as the count of zero crossings, must be equal to each other or different by at most one [26].
2. The average of the envelope (defined by the local maxima and local minima) calculated in the EMD algorithm (see Algorithm 18) should be zero at any time point [26].

The dataset used in this study was taken from the MIT/BIH arrhythmia database, and it consisted of signals that were classified as the following: normal, left, and right bundle branch blocks, premature ventricular contraction, paced beat, and atrial premature beats. 27 records were selected, and each record contained 2-channel ECG signals, with a duration of 30 min each. A sampling rate of 360 Hz was used, and the signals were bandpass filtered between 0.1 and 100 Hz [26].

**Table:** 

**Algorithm 18: Empirical Mode Decomposition **[26]	
1. **Result**: IMF signals, PSD features, and variance of PSD features	
2. Import the ECG signal	
3. Preprocess the ECG signal	
a. 10th-order Butterworth low-pass filter with 53 Hz cut-off frequency	
b. 3rd-order Butterworth high-pass filter with 0.75 Hz cut-off frequency	
c. Bandpass filter between 0.1 and 100 Hz	
4. Perform EMD to decompose signal into IMFs (7)	
a. Find the local maxima and local minima of the original signal (x(t))	
b. Generate the upper and lower envelope. Use a cubic spline interpolation between the extrema points	
c. Average the upper and lower envelope	
i. $m(t) = [{e}_{min}(t)+{e}_{max}(t)]/2\quad \quad (15)$	
d. Subtract the average from the original signal	
i. $h(t)=x(t)-m(t)\quad \quad (16)$	
e. Check if result meets the requirements of IMF	
i. If yes, then the IMF is formed. Go to f)	
ii. If not, repeat the procedure	
f. Subtract IMF from the original signal to find the residual signal	
i. ${r}_{1}(t)=x(t)-IMF\quad \quad (17)$	
g. Repeat steps a–f using the previous residual function found as the original signal x(t)	
h. Repeat until the residual signal calculated is a monotonic function	
5. Perform feature extraction on the original signal and IMF1-IMF7	
a. Feature 1: Power Spectral Density (PDF)	
b. Feature 2: Variances of PDF	
6. Use features from the original signal, IMF1 and IMF2 for classification	

### The wavelet transform (WT)

The wavelet transform (WT) is used as it can provide excellent localization in the time and frequency domains simultaneously [8]. For a signal *f(t)* with a mother wavelet of *ψ(t)*, the WT is defined as follows:

18 $${W}_{f}(a,b) = <f,{\psi }_{a,b}> = {\left|a\right|}^{-0.5}{\int }_{r}f(t)\psi (\frac{t-b}{a})dt$$

Note that *a* is the dilation factor and *b* is the translation factor. Changing these parameters achieves different frequency and time localizations [27]. Since the ECG signal is discretized, we must also discretize the wavelet transform. The discrete wavelet function is defined as the following [27, 28]:

19 $${\psi }_{m,n}(t)= {{a}_{0}}^{-\frac{m}{2}}\psi (\frac{t-n{a}_{0}^{m}{b}_{0}}{{a}_{0}^{m}})={{a}_{0}}^{-\frac{m}{2}}\psi ({a}_{0}^{-m}t-n{b}_{0})$$

The dataset used in the studies examined for the wavelet transform used the MIT/BIH database. Please note that typically the continuous wavelet transform is used for feature extraction applications, while the discrete wavelet transform is used for data compression applications. This is because there are complications with the discrete wavelet transform due to its nature of time variance [3].

**Table:** 

**Algorithm 19: Wavelet Transform Algorithm **[27]	
1. **Result**: WT decomposed ECG signal	
2. Import the ECG signals	
3. Preprocess the ECG signals	
a. Filter to remove the high-frequency noise and baseline drift	
4. Segment the signal into 5-s duration samples	
a. Perform R-peak detection for each of the segments	
i. Calculate the R-R intervals	
b. Compute mean and variance of R–R intervals (features 1 and 2)	
5. Segment the signal once again, this time based on the R peaks to obtain single-period waveforms	
6. Perform WT to decompose the signals	
a. Reconstruct the characteristic waveform using the decomposition coefficients from the fourth layer	
7. Compute FFT of the characteristic waveform	
a. Obtain the maximum amplitude in the frequency spectrum (feature 3)	

### Singular value decomposition (SVD)

SVD is popular since it can be used for data compression of ECG signals, while also being used to extract significant feature components of the ECG. SVD decomposes the ECG signal into sets of basic patterns with their own scaling factors; then, only the relevant parts of the singular triplets would be required to be retained to retrieve the original signal, resulting in data compression [29].

To perform SVD, first the signal needs to be rearranged as a 2D matrix. Considering that the signal is periodic, and has m consecutive periods,

20 $$A= \left\{{x}_{i}\left(t\right)\left|i=1,\dots ,m;t=1,\dots ,n\right.\right\}=\left[\begin{array}{ccc}x(1)& \cdots & x(n)\\ \vdots & \ddots & \vdots \\ x(\left(m-1\right)n+1)& \cdots & x(mn)\end{array}\right]$$

Then, SVD can be performed using the following, considering U and V are the left and right singular vectors, respectively [29].

21 $$A = U\sum {V}^{T}$$

From A, which is composed of the repetitive pattern of consecutive rows, can be decomposed into the basic patterns of the signal, which can also be used to reconstruct the signal. The less significant singular values will be eliminated, which consequently performs data compression [29].

**Fig. 9:**
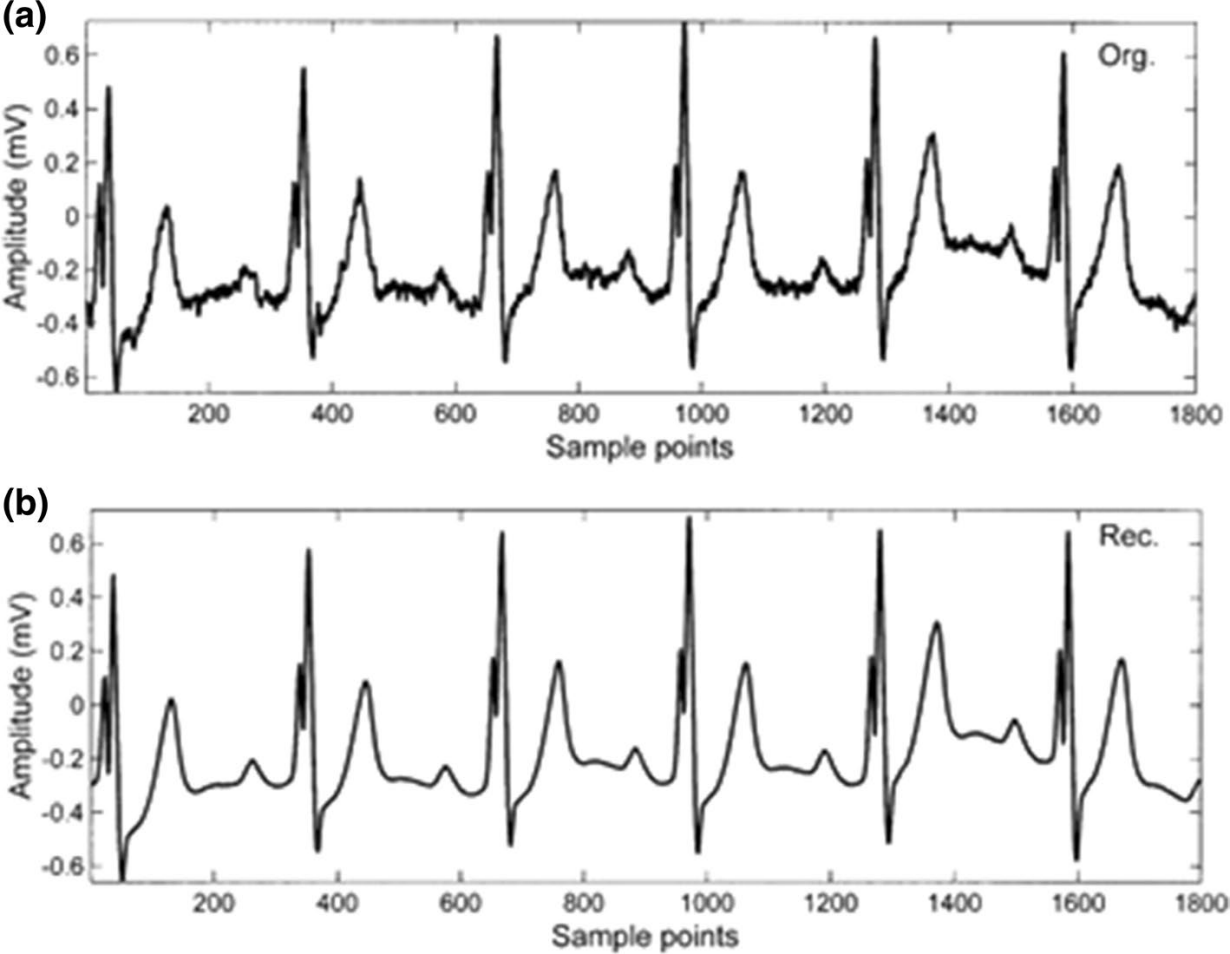
**a** shows the original signal, and **b** shows the reconstructed signal using the SVD algorithm [29]

The dataset from the MIT/BIH database was used in this case study. The data consisted of ten-minute data records, sampled at 360 Hz. Different ECG rhythms were chosen to evaluate the reconstruction aspect of the algorithm. Refer to Fig. 9 displaying the original signal (a) and the reconstructed signal using the SVD algorithm (b). Note the similarity in shape; this shows that the features extracted from the SVD algorithm carry an appropriate degree of the underlying signal data, allowing for accurate reconstruction [29].

**Table:** 

**Algorithm 20: SVD Algorithm **[29]	
1. **Result**: Features: R-R interval, Mean Beat Period (MBP)	
2. Import ECG Signals	
3. Preprocess the ECG signals	
4. Perform beat delineation (QRS detection) for periodic segmentation	
a. Store R-R interval information	
5. Normalize the segmented ECG cycles to the same periodic length	
a. MBP is chosen as the normalized length	
6. Perform SVD transformation, using Eqs. 20 and 21	
7. Perform reconstruction using the features extracted	

### Intrinsic Time-Scale decomposition (ITD)

Intrinsic time-scale decomposition (ITD), is popularly used to de-noise ECG signals. This would lead to more effective feature analysis for a variety of applications, such as arrhythmia classification and detection; note that the case study analyzed did not focus on feature extraction, but it can definitely be expanded on [30].

During the acquisition and transmission of ECG signals, the signal is impacted by noise, such as Gaussian noise, powerline interference, muscle artifacts, and baseline wander. There are filtering techniques that could be employed, but they do not preserve the low-frequency ECG components of the signal [30].

Please note that in the following equations, x(t) is the original signal, L is an operator used to extract a baseline signal from x(t) to form a proper rotation, H. The baseline signal is Lt. [30].

22 $$x(t) = Lx(t) + (1-L)x(t) = {L}_{t}+H$$

23 $${L}_{t}=Lx(t)$$

24 $$H = (1-L)x(t)$$

When ITD is performed, the signal is broken down into components. In traditional ITD algorithms, to remove the noise, the noisy components of the signal are simply disregarded. This is also helpful for data compression. The lower order components (1–3) contain the high-frequency information, like the QRS complex, and the noise. In this method however, we will perform wavelet-based denoising, as we want to preserve the QRS complexes, not reject them. Steps to compute the wavelet denoising of a signal have been outlined in depth in [30].

The dataset used in this study was taken from the MIT/BIH database; 6 ECG records were used, all of 30 min durations each. Electromyography (EMG) noise was added to the signals to analyze the denoising power of the algorithm. The signal-to-noise ratio (SNR) is used to evaluate the performance [30].

**Table:** 

**Algorithm 21: ITD algorithm **[30]	
1. **Result**: ITD decomposed ECG signal	
2. Import noisy ECG signal	
3. Perform ITD to decompose the signal into 8 components + a residual signal	
a. Use Eqs. 22–24	
4. Perform wavelet denoising of the components. Refer to “Generalized Tensor Rank One Discriminant Analysis (GTR1DA)” Section	
5. Reconstruct the signal	
6. Perform R-peak detection, and extract theirlocations as features	

### Matching pursuits (MP)

**Fig. 10:**
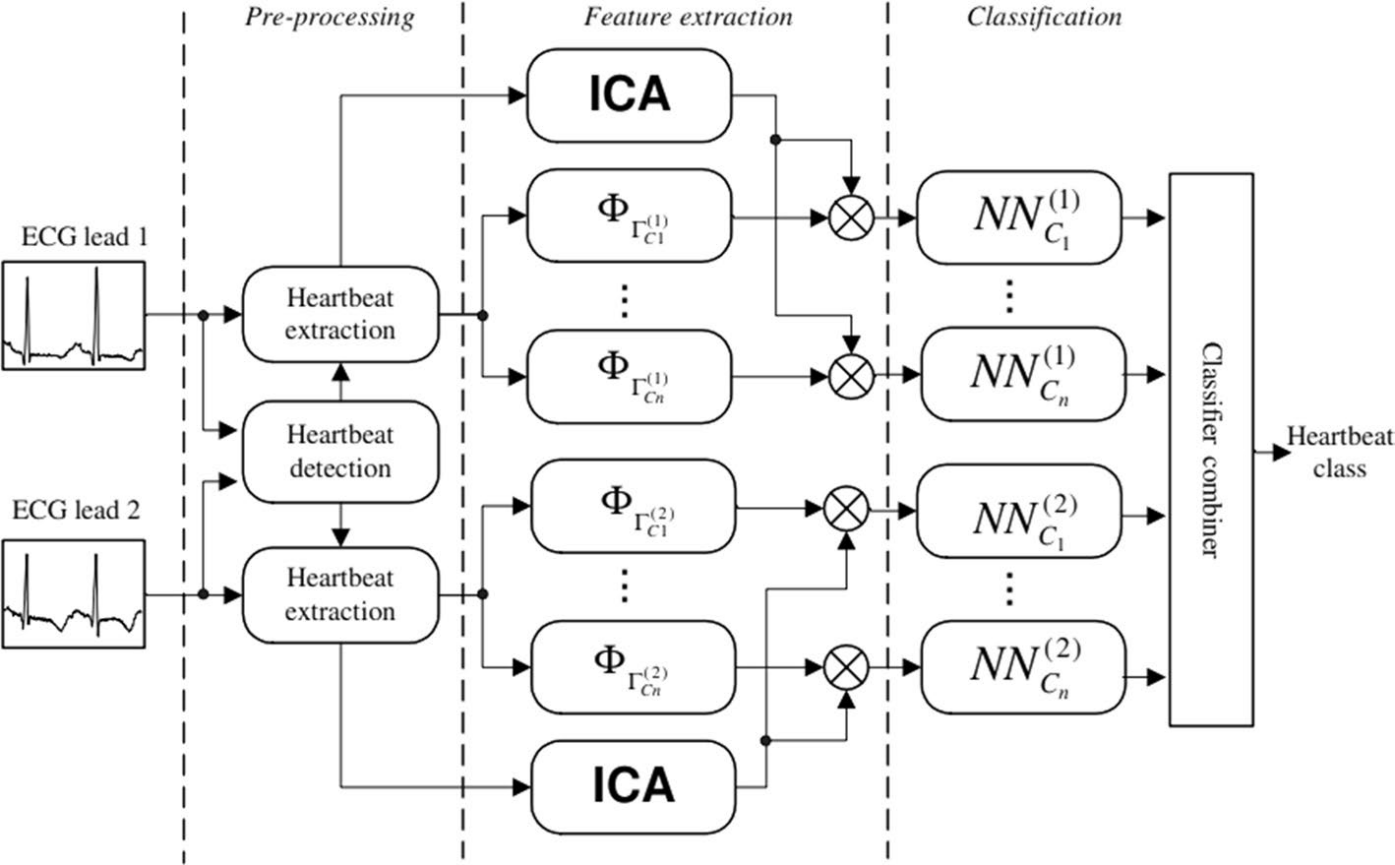
Proposed system for MP and ICA feature extraction [33]

The matching pursuits (MP) algorithm is used for extracting time–frequency features for classification of abnormal heartbeats in the case study analyzed; essentially, the MP algorithm is used to select the time–frequency basis that is optimal for the detection of different beat patterns. This algorithm is further augmented with the use of independent component analysis (ICA) for extracting spatial features; ICA is a statistical technique, similar to PCA which was discussed in “Time-domain Feature Extraction” Section. Essentially, each heartbeat will be projected into different wavelet packet sets that are selected based on the matching of characteristic structures of the different beats that the algorithm is attempting to classify. Wavelet packets are used due to their high localization power. Refer to Fig. 10 for the proposed MP and ICA feature extraction system [31].

Let *s(t)* be the original noisy signal. *D* is the dictionary of waveforms ${{(\phi }_{\gamma })}_{\gamma \epsilon \Gamma }$ where γ is the indexing parameter of *D*. The decomposition of *s(t)* can be approximated by the following [31]:

25 $$s\left(t\right)= {\sum }_{i=1}^{m}{\alpha }_{\gamma i}{\phi }_{\gamma i}+{r}^{\left(m\right)}$$

where *r* is the noise that is being separated from the original signal.

The dataset used in this study was taken from the 48 two-lead ECG recordings available in the MIT/BIH database. Each recording is 30 min in duration and the sampling frequency is 360 Hz [31]. Please refer to Table 4 for the summary of the decomposition domain feature extraction methods discussed.

**Table 4:** Summary of decomposition domain feature extraction methods for ECG

Method	Advantages	Disadvantages	Sample applications
Empirical mode decomposition	Signals are processed in the time domain and thus it is easier to evaluate the analysis EMD removes the high-frequency components and keeps the useful information of ECG signals to extract efficient features	Only achieved 87% accuracy. Features chosen may not be the most appropriate to achieve a high accuracy—more research is needed	Arrhythmia detection [26]
Wavelet transform	Excellent localization in the time and frequency domains simultaneously	The presence of some arrhythmias may lead to inaccurate detection of QRS complexes [8] Applying 3-lead systems of ECG signal may cause the loss of signal information which could limit efficiency of the WT [8]	Arrhythmia detection [27, 28]
Singular value decomposition	Allows for data compression High computational efficiency Provides efficient coding with high-compression ratios Noise-filtering capability	Noise filtering of original signals in SVD-based approach contribute to a relatively larger error in the reconstructed signals	Data compression and recoverability of ECG signals [29]
Intrinsic time-scale decomposition	Reduces noise in the QRS regions and enhances the QRS complexes Efficient decomposition into “proper rotation: components; the frequency and amplitude are well defined	Higher computational power required for more complex signals Features may not always be accurate	Denoising ECG signals [30]
Matching pursuits	High discrimination performance Approach is more flexible than other leading approaches	Higher degree of features required for the algorithm The algorithm can be optimized, at which point the performance would improve Does not perform well with the PVC class of heartbeats High computing power required Overall is a greedy algorithm for approximating decompositions	Heartbeat Classification [31]

**Table:** 

**Algorithm 22: Matching Pursuits Algorithm **[31]	
1. **Result**: Decomposition of ECG signal	
2. Import ECG signal	
3. Preprocess the ECG signals	
a. Bandpass filtered from 0.1 to 100 Hz	
4. Perform heartbeat detection	
a. Use the fiducial points of the database and extract samples using a fixed window around each fiducial point	
5. Denoise the sets of heartbeats using the MP algorithm:	
$$\hat{S}\,\left( 0 \right) = 0$$	
$$r\left(0\right)=s$$	
$$i=1$$	
$$while \left(i\le m\right)$$	
$${\gamma }_{i}={argmax}_{\gamma }{\Vert {r}^{(i-1)},{\phi }_{\gamma }\Vert }_{1}$$	
$${\alpha }_{\gamma i}= \langle {r}^{\left(i-1\right)},{\phi }_{\gamma i}\rangle$$	
$$\widehat{S}\left(i\right)=\widehat{S}\left(i-1\right)+{\alpha }_{\gamma i}{\phi }_{\gamma i}$$	
$$r\left(i\right)=S-\widehat{S}\left(i\right)$$	
$$i=i+1$$	
$$end$$	
6. Perform ICA feature extraction	
7. Select the *m* wavelet packet atoms that best match the structures in each class	
8. Compute the average normal of the wavelet packet atoms	
a. Rank the average norms	
i. The atoms with the greatest average represent the most important/stable signal structures	
9. Use the features extracted to train a machine learning model	

## Deep learning

Deep learning (DL) is a subset of machine learning (ML), which was discussed in “Significance of Features for Machine Learning” Section*.* Essentially, DL attempts to mimic human interaction using a neural network of 3 or more layers. DL and the features it uses can be used to drive artificial intelligence (AI) to perform tasks without manual intervention. DL differs from ML because it does not require the data pre-processing that is needed with ML; this means, DL is still efficient when it is faced with unstructured data, and does not require input from human experts, like supervised ML models do. DL automatically detects features that are most useful for classification purposes [32].

The underlying neural networks are made up of multiple layers of interconnected nodes which work to refine and optimize the task at hand. There are a variety of deep neural networks available for use, such as convolutional neural networks (CNNs) and recurrent neural networks (RNNs). The network is chosen based on the application or problem that the developer is trying to solve [32].

DL is being applied in the healthcare domain as well; one case discusses the application of a CNN model to the ECG for classification of heart disorders. First, automatic feature extraction is performed from the data inputted. In the next step, the fully connected, multi-layer perceptron works to classify what was learned in the first step [33].

The dataset used in this study was taken from the Physikalisch-Technische Bundesanstalt Diagnostic ECG records from the Physionet database consisting of 549 ECG signals with normal and abnormal recordings. Although DL is meant to eliminate the pre-processing of the data, this case study performed some filtering. Please refer to Algorithm 23 for a summary of the process. The classification results were in the high-80 range in terms of performance; Accuracy = 88.33%, Sensitivity = 89.47%, and Specificity = 87.80%. Performance can be improved with the use of other DL methods, which goes back to the point that the choice of DL method is heavily dependent on your application and/or problem you want to solve [33].

**Table:** 

**Algorithm 23: Deep Learning—CNNs **[33]	
1. **Result**: DL pipeline	
2. Import ECG signals	
3. Pre-process ECG signals	
a. Symlet scaling filter from the wavelet transform	
b. Savitzky–Golay filter	
4. Use AlexNet from the CNN architecture family	
a. For performing feature extraction before moving onto the classification stage	
5. Use the features in an ML model—> Extreme Learning Machine (ELM) model	
a. Use the ELM sigmoid function as it provides the best results	

## Discussion, conclusion, and future work

Through this review, we have studied various methods of feature extraction from the time domain, frequency domain, time–frequency domain, and the decomposition domain. As we progress through these stages, the signal data dimensions are observed to increase, but signal representation via the features is also improved through the domains. It was also analyzed in “Significance of Features for Machine Learning and Deep Learning” Section, that the features can be integrated into ML pipelines for the various applications discussed in Tables 1, 2, 3, 4. Note that a practical feature extraction pipeline needs to generate robust features, compress the underlying data through dimensional reduction, and be easily integrated with an ML model [4].

Although the time domain and frequency domain features are fairly straight forward to extract from signals, on their own, they do not optimize representation of the underlying signals. This is critical to achieve when applying feature extraction methods to physiological signals. Hence it was determined that the time–frequency domain methods perform better for ECG applications. This is primarily due to the non-linear and non-stationary characteristics that ECG signals carry [3].

Popular methods in the time domain, like LPC, have been shown to have fast processing, but again face the issue of the LPC coefficients not carrying enough information about the ECG signals for a robust analysis. Hidden Markov models have high computational complexity, so it would be difficult to apply this for real-time systems. This is yet another challenge faced by engineers; developing pipelines that are computationally simple enough that a result can be outputted in real-time for quick analysis. This is a tradeoff that is observed. For some of the more accurate methods, they are more computationally expensive, and take more time to actually run and compute. But as engineers, this is a design choice that needs to be carefully considered and weighed depending on the problem that you are trying to solve.

In the frequency domain, methods, such as the Hilbert transform, FFT, DFT, MFCC, and the DCT, are all very accurate and precise for their applications. This is further solidified as we know the frequency component of ECG signals is highly important for analysis. Hence, frequency features would intuitively result in a better representation of the data (when compared to the time domain methods), and thus report greater results. However, these methods also have their drawbacks as they are sensitive to noise, and we sometimes see difficulty when considering scaling the methods for multi-lead ECG signals. Many of the methods commonly used are for single or two-lead ECGs.

Moving onto more complex solutions, the time–frequency domain combines the concepts from the previous two domains. These methods provide more insight into the frequency components with time instances associated as well. This would further increase the accuracy and representation of the ECG signals, which would theoretically lead to better results. We do see excellent results with the methods discussed in this review. For example, the WVD has excellent resolution for the energy along both the time and frequency axes of the signals. The TR1DA method is helpful because it can represent the signal without compromising structural information like other methods do. Although CWD has high accuracy, it should be noted that with these methods, there is a tradeoff with time and frequency resolution; this means it is not possible to have both optimized, and some of these methods can also be computationally exhaustive. Again, this will need to be carefully considered by the practitioner developing the algorithm, and it must be justifiable as a design choice.

The decomposition domain is useful as it allows for the decomposition of the ECG signal, after which the irrelevant components can be disregarded, and the desired components can be accepted. This can also be used to pre-process signals as the noisy components can be removed. Furthermore, this also results in data compression and can be applied to the IoT and healthcare domains.

Some of the methods discussed in this review are EMD, WT, SVD, and MP. However, there are drawbacks to these methods. These methods require signal approximation, which results in information loss. The result is lower feature accuracy, larger errors. These methods also require higher computational power. [3, 26–29, 31]. Typically, in the decomposition domain, to combat the drawbacks, a larger degree of features is required to represent the underlying data.

**Fig. 11:**
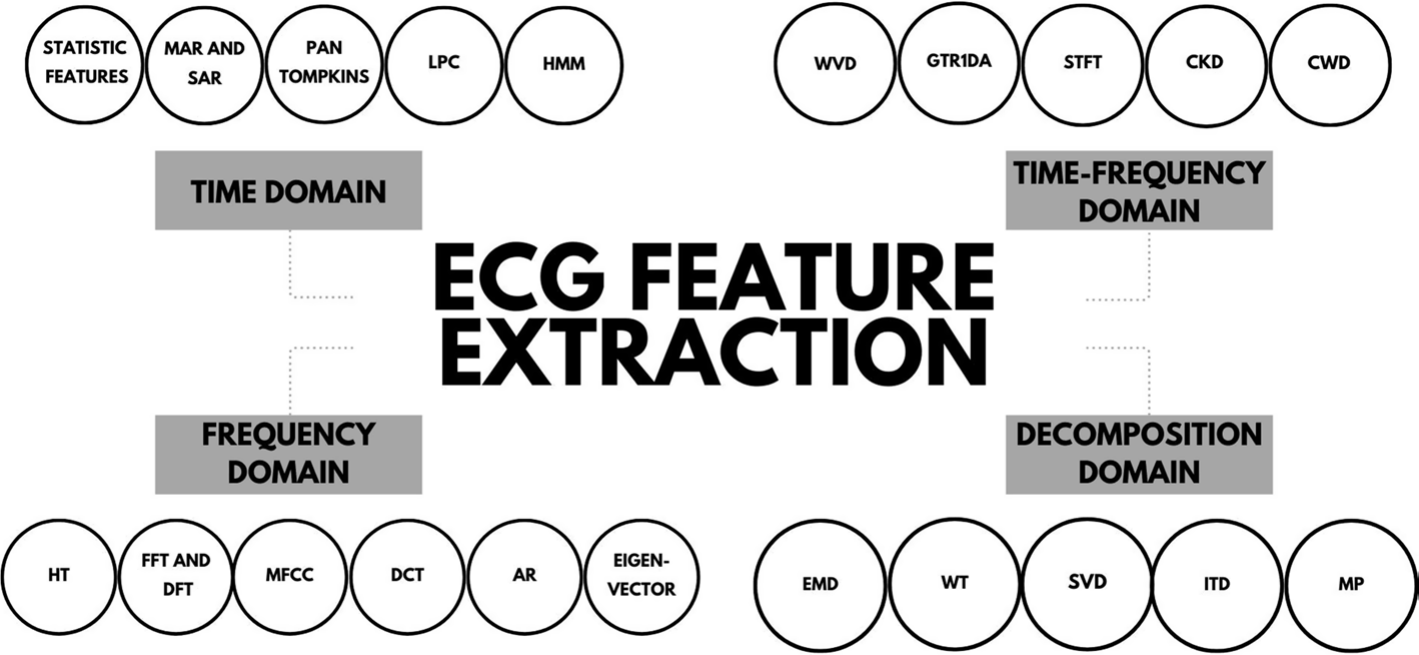
Quick reference of ECG feature extraction methods

In this review, we focused on methods to compare their computational complexity, data compression capabilities, robustness and accuracy of features extracted, and handling of non-linearity and non-stationarity. Each method, summarized in Fig. 11 has its own sets of pros and cons (refer to Tables 1, 2, 3, 4), which will need to be weighed by the reader during implementation and testing. Based on the analysis of the various methods, it is clear that time–frequency provides the best representation on average of ECG signals. However, it still is important to consider the methods in the other domains depending on the application or problem that you are trying to solve.

There is definitely room for future work and research with the feature extraction methods. Many of the methods discussed can either be applied to single-lead ECGs or multi-lead ECGs. Recall that, traditionally, a 12-lead ECG is taken, but for more modern applications, single-lead ECGs are becoming more desirable and commonplace due to the reduction of complexity and data. This can be applied to the IoT and connected healthcare domains. However, multi-lead ECGs are used more in clinical settings because it is the gold standard; single-lead/reduced-lead ECG signals are not typically accepted in the primary healthcare and clinical workspaces. Majority of the methods discussed are applied to single or 2-lead ECG signals, meaning that the results would not be clinically accepted. Thus, there is room for research here, where perhaps the solutions can be scaled in such a way that the primary healthcare system can benefit.

Also, there is room for improvement for extending the solutions discussed in this review for real-time applications. As the health-technological revolution continues, we will be required to innovate in this regard. The real-time systems need to be improved so that the accuracy of the results rival that of the more robust yet computationally expensive methods such that they can be clinically accepted methods in future. There are a wide variety of challenges that this could solve, including reducing some of the strain that the healthcare system faces due to the backlog of patients. Real-time solutions would allow for the processing of more patients in a shorter period of time.

## Data Availability

Data sharing is not applicable to this article as no datasets were generated or analyzed during the current study.
